# Regional left ventricular deformation in primary mitral regurgitation

**DOI:** 10.1186/1532-429X-13-S1-P336

**Published:** 2011-02-02

**Authors:** Yuchi Han, Susan B Yeon, Idith Haber, Kraig V Kissinger, Beth Goddu, Warren J Manning

**Affiliations:** 1 BIDMC, Boston, MA, USA

## Introduction

MVP is a common disorder affecting 2-3% of the general population. MVP patients with severe MR may remain asymptomatic for a number of years; however, LV systolic function may be impaired post-operatively, even among those with normal pre-operative ejection fraction (EF). Myocardial strain is the assessment of myocardial local deformation and may provide a more accurate assessment of LV function.

## Purpose

We sought to study the effect of volume overload on the left ventricle (LV) imposed by increasing mitral regurgitation (MR) by analyzing LV circumferential myocardial strain, peak systolic strain rate (sSR), and peak early diastolic strain rate (dSR) using myocardial tagging in mitral valve prolapse (MVP) patients with MR severity ranging from mild to severe.

## Methods

CMR studies were performed on a 1.5 T Philips Achieva MRI scanner (Philips HealthCare, Best, NL), equipped with a 5-element cardiac coil. Short axis breath-held balanced SSFP sequences for LV function and phase-contrast cine in the axial plane for aortic flow were performed. MR volume = LV stoke volume - aortic flow. MR fraction = MR volume/stroke volume and graded from mild to severe. Tagged MRI images were acquired using 2D CSPAMM sequence at the LV mid papillary muscle level and analyzed using Cardiotool (MATLAB, Mathworks, Natick, MA) for myocardial circumferential strain. The slices are divided into six segments.

## Results

**Figure 1:**
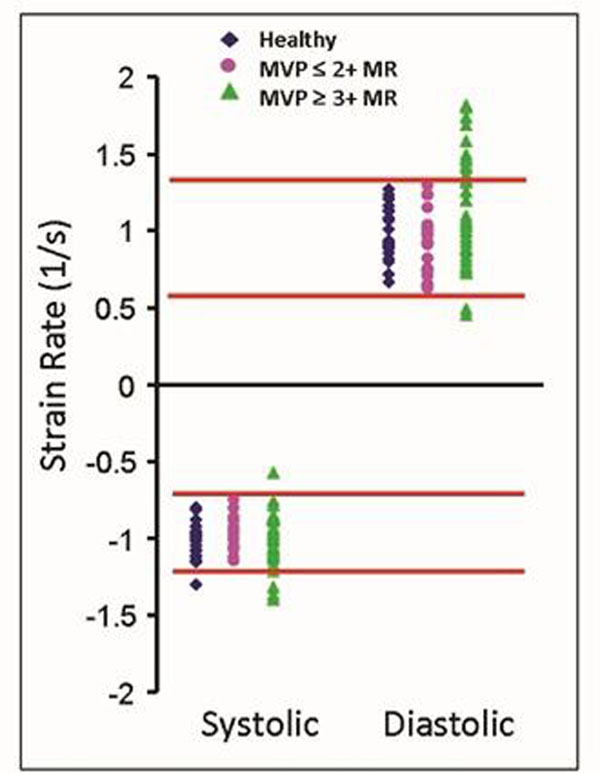
**Compare healthy control, mild to moderate MR subjects and moderate to severe MR subjects by peak systolic strain rate and peak diastolic strain rate** (red line denote the range of control and subject with mild MR). Standard deviation (heterogeneity) in both parameters is increased in subjects with severe MR.

Of the 65 MVP patients (age 52 ± 11 years, 60% males, LVEF 64 ± 5%) studied, 23 had ≤2+ MR (15± 8%) and 43 had ≥3+ MR (41± 9%). There was no significant difference in LVEF (p= 0.17). 25 healthy adult subjects served as controls (age 33 ± 14 years, 56% males, LVEF 63 ± 3%). Compared to controls, both MVP groups had reduced systolic strain and sSR in the inferolateral segment (p < 0.002) and increased dSR in the anterior segment (p<0.001). Compared to patients with mild MR, patients with severe MR have increased dSR in the anterior segment (p<0.001). The distribution of sSR and dSR in the control and mild MR group is similar (Figure 1) while the severe MR group has increased heterogeneity, particularly in dSR.

## Conclusions

Regional myocardial deformation offers additional information regarding LV function in primary MR than LVEF. The role of strain parameters in patient assessment needs to be clarified in longitudinal follow up studies and pre and post surgical evaluations.

